# Hospital-based patterns of esophageal and gastric cancer cases at a major tertiary referral center in Kabul, Afghanistan: a retrospective case series

**DOI:** 10.1186/s12885-026-15945-z

**Published:** 2026-03-29

**Authors:** Mohammad Farid Tawakoli, Hamed Salihee, Abdulhafiz Rahmati, Mohammad Nasim Hatami

**Affiliations:** Public Health Faculty, Kabul University of Medical Sciences, Kabul, Afghanistan

**Keywords:** Esophageal cancer, Gastric cancer, Hospital-based study, Case series, Afghanistan, Risk factors

## Abstract

**Background:**

Esophageal and gastric cancers are leading causes of cancer-related mortality worldwide, with a disproportionate burden in low and middle-income countries. In Afghanistan, population-based cancer data are scarce, and hospital-based studies remain essential for understanding local disease patterns. This study aimed to describe the demographic characteristics, histopathological patterns, and distribution of esophageal and gastric cancer at a major tertiary referral center in Kabul.

**Methods:**

This retrospective hospital-based case series included all patients diagnosed with esophageal or gastric cancer at Jumhoriat Hospital, Kabul, between October 2023 and August 2024. Demographic, socioeconomic, behavioral, and histopathological data were extracted from medical records. Descriptive statistics were used to summarize findings, and the chi-square test assessed the association between sex and cancer type.

**Results:**

Out of 3250 registered cancer cases, 917 patients were diagnosed with esophageal or gastric cancer. Esophageal cancer accounted for 708 cases (77.2%), while gastric cancer comprised 209 cases (22.8%). The highest proportion of cases occurred in the 55–64-year age group for both esophageal (28.2%) and gastric cancers (32.0%). Esophageal cancer showed a relatively balanced sex distribution (53.2% male), whereas gastric cancer was significantly more frequent among males (71.2%) (χ² = 20.75, *p* < 0.001). Squamous cell carcinoma was the predominant histological subtype of esophageal cancer (70%), while adenocarcinoma was the most common subtype of gastric cancer (81.3%). A high proportion of patients, particularly those with esophageal cancer (85.3%), belonged to a low economic status, and tobacco use was frequently reported among patient.

**Conclusions:**

Esophageal cancer constitutes the majority of upper gastrointestinal cancer cases at this major referral center in Kabul, with both malignancies predominantly affecting individuals aged 55–64 years. The observed patterns align with reports from other low-resource settings and highlight the need for targeted prevention strategies, strengthened early detection, and investment in a population-based cancer registry in Afghanistan.

## Introduction

Esophageal and gastric cancers are among the most important malignancies of the gastrointestinal tract and represent major contributors to cancer-related morbidity and mortality worldwide. Global epidemiological reviews consistently report a substantial burden of these cancers, particularly in low- and middle-income countries, where late diagnosis and limited access to specialized healthcare services remain common challenges [1–3]. According to the most recent estimates from the Global Cancer Observatory (GLOBOCAN 2022), approximately 510,716 new cases and 445,179 deaths were attributed to esophageal cancer worldwide, ranking eleventh in incidence and seventh in cancer-related mortality. Gastric cancer accounted for an estimated 968,350 new cases and 659,853 deaths, ranking fifth in both incidence and mortality globally. Notably, a disproportionate share of this burden occurs in Asia countries, where both incidence and mortality rates remain comparatively high [4].

The epidemiology of esophageal cancer shows marked geographical variation, with high-incidence regions reported across parts of Central and South Asia. This geographic clustering, often described as an esophageal cancer belt, has been attributed to a combination of environmental, lifestyle-related, and population-specific factors rather than a uniform global distribution [1, 5, 6]. Northern Afghanistan has been included in studies focusing on these high-risk regions, with hospital-based investigations reporting a notable frequency of esophageal cancer among patients evaluated for upper gastrointestinal [7, 8].

In addition to geographic variation, differences in esophageal cancer occurrence among ethnic groups living within the same regions have been reported. Studies from northern Afghanistan indicate higher frequencies among certain ethnic populations, including Uzbek and Turkmen groups, suggesting potential associations with environmental exposures and lifestyle practices specific to these communities [5, 8].

Gastric cancer also remains a major global public health concern. Although declining trends have been observed in some regions, gastric cancer continues to contribute substantially to cancer-related mortality, particularly in Asian countries. Epidemiological reviews emphasize that late-stage diagnosis remains common and that the disease burden is still considerable in many low-resource settings [9–11]. Regional evidence from neighboring countries, especially Iran, has documented significant variation in gastric cancer patterns and has highlighted the relevance of infectious, dietary, and lifestyle-related factors within similar environmental and cultural contexts [10, 12].

Across global and regional studies, both esophageal and gastric cancers demonstrate consistent demographic patterns, with higher frequencies reported among males and older age groups. These trends have been widely documented and remain evident despite regional differences and temporal changes [3, 6, 13].

Hospital-based studies from Afghanistan have further elucidated these patterns. A recent analysis from the oncology department of Jamhuriyat Hospital, the primary tertiary referral center in Kabul, reported on 1025 cancer patients diagnosed between October 2015 and December 2017 [14]. This study demonstrated that esophageal was the most common malignancy among men, accounting for 21.8% of male cases, followed by stomach cancer (12.2%). Among women, esophageal cancer was the second most common cancer (12.5%), following breast cancer [14]. The median age for esophageal cancer patient was 58 years, while stomach cancer showed a male predominance and higher frequency in older age group [14]. These findings align with the regional pattern of Afghanistan’s location within the Central Asian Esophageal Cancer Belt and confirm that squamous cell carcinoma remains the predominant histopathological subtype in this population with most patients presenting in middle to older age groups [14].

In Afghanistan, particularly in Kabul, population-based cancer registry data are scarce. Available evidence on esophageal and gastric cancers is largely derived from hospital-based studies conducted in tertiary referral centers such as Jumhoriat Hospital, making institutional case series essential for describing local disease pattern. This study represents one of first institutional analyses from Kabul following the health care system transitions since 2021, providing and updated disease landscape. Furthermore, it seeks to determine whether the previously reported predominance of squamous cell carcinoma for esophageal cancer and adenocarcinoma for gastric cancer persists in the current patient population. Therefore, this study aimed to describe the frequency, distribution, and demographic and histopathological patterns of esophageal and gastric cancer cases diagnosed at Jumhuriat Hospital, a major tertiary referral center in Kabul, Afghanistian.

## Methodology

### Study design and setting

This study was a hospital-based, retrospective descriptive case series conducted to describe the frequency, distribution, and histopathological patterns of esophageal and gastric cancer cases. The reporting of this study follows the Strengthening the Reporting of Observational Studies in Epidemiology (STROBE) guidelines for observational studies.

The research was carried out at Jumhoriat Hospital, a major tertiary referral center located in Kabul, Afghanistan. Jumhoriat Hospital is recognized as one of the primary specialized oncology centers in the country and serves as the main referral hospital for cancer patients from Kabul province as well as from all other provinces of Afghanistan [14]. The hospital’s oncology department provides basic diagnostic services including endoscopy, pathology, and radiology for histopathological confirmation and staging of upper gastrointestinal malignancies. However, similar to other low-resource settings, the hospital faces limitations in terms of advanced diagnostic equipment and specialized oncology workforce, which may impact the completeness of diagnostic documentation [14].

### Study population and period

The source population comprised all patients with a cancer diagnosis whose medical records were registered in the oncology ward of Jumhoriat Hospital between October 2023 and August 2024. From this source population, all cases with a confirmed diagnosis of either esophageal or gastric cancer were included in the final study sample.

### Data source and data collection

Data were collected retrospectively through a systematic review of patients’ medical records (dossiers) using a structured data extraction form. The medical records were reviewed by the authors. The data extraction form was designed to capture the following variables:

- Demographic characteristics: Age, sex, ethnicity, marital status, and province of residence.
- Socioeconomic and behavioral variables: Occupation, economic status, and history of tobacco use.
- Clinical and anthropometric data: History of chronic disease and Body Mass Index (BMI).
- Diagnostic and histopathological data: Cancer typ (esophageal/gastric), diagnostic modality (endoscopy, radiology), and final histopathological diagnosis (e.g., squamous cell carcinoma, adenocarcinoma).

To ensure data quality, the extracted information was checked for completeness and consistency. Any records with ambiguous or illegible entries were cross-verified by a second reviewer. Cases with missing essential data (age, sex, or definitive diagnosis) were excluded from the analysis.

### Definition of key variables

To ensure clarity and reproducibility, the following operational definitions were applied:

#### Economic status

This was classified as “good” or “weak” based on the patient’s self-reported ability to cover basic living expenses and medical costs, as documented in the medical records by the attending physician during the initial history taking. In the absence of standardized income metrics in Afghanistan, this classification relied on the physician’s assessment documented in the patient’s file, similar to the approach used in previous hospital-based studies in this setting [14].

#### Tobacco use

Tobacco use was defined as the use of tobacco products, including cigarettes, cigars, pipe tobacco, chewing tobacco, and naswar. Information regarding tobacco use was extracted from the habitual history section of the medical records. Smoking status was recorded based on documented history of tobacco consumption.

#### Chronic disease history

This was defined as the presence of any long-term medical condition (e.g., hypertension, diabetes, cardiovascular disease) documented in the patient’s past medical history.

#### Body Mass Index (BMI)

BMI was calculated as weight in kilograms divided by the square of height in meters (kg/m²) and categorized according to WHO standards (underweight: <18.5, normal: 18.5–24.9, overweight: 25-29.9, obese: ≥30). data, BMI was recorded as “unknown”.

### Sampling method

A consecutive sampling method, specifically a census approach, was employed. All medical records of patients diagnosed with esophageal or gastric cancer during the defined study period that met the inclusion criteria were included in the study. No formal sample size calculation was performed, as the aim was to include the entire accessible population.

During the study period, a total of 3250 cancer cases were registered at the hospital. After applying the eligibility criteria, 917 records of patients with esophageal or gastric cancer were identified and constituted the final study sample.

### Inclusion criteria

- Medical records of patients with a confirmed diagnosis of esophageal or gastric cancer.
- Records registered within the study period (October 2023 to August 2024).
- Records containing sufficient information on at least the primary diagnosis and demographic data (age and sex).

### Exclusion criteria

- Medical records of patients with primary cancer types other than esophageal or gastric.
- Records with incomplete or missing key pages that prevented the extraction of the primary diagnosis.
- Duplicate records or records registered outside the specified study period.
- Records with missing key clinical or histopathological data (age, sex, or cancer type).

### Statistical analysis

Data were entered into Microsoft Excel, coded, and subsequently analyzed using IBM SPSS Statistics (Version 26). Descriptive statistics, including frequencies and percentages, were calculated to summarize the demographic, socioeconomic, clinical, and histopathological characteristics of the study population.

To examine the association between categorical variables, specifically the relationship between sex (male/female) and cancer type (esophageal/gastric), a Chi-square (χ²) test of independence was performed. A p-value of less than 0.05 was considered statistically significant. Due to the descriptive and retrospective nature of the case series design, no multivariable or causal inferential analyses were conducted.

### Handling of missing data

A substantial proportion of patients (approximately 38%) had missing BMI data, primarily due to incomplete documentation of height or weight in the medical records. These cases were categorized as “unknown” in the analysis, and no imputation methods were applied. Similarly, for other variables with occasional missing values, only complete cases were analyzed, and the proportion of missing data is clearly indicated in the tables.

### Ethical considerations

This study was conducted in accordance with the Declaration of Helsinki. Official administrative permission was obtained from the Public Health Ethics Committee, Kabul University of Medical Sciences (Abu Ali Ibn Sina) to review the medical records at Jumhoriat Hospital (permission letter No: 344).

As this was a retrospective review of existing medical records with no direct patient contact or intervention, the requirement for informed consent was waived by the ethics committee. To ensure patient confidentiality, all extracted data were fully anonymized prior to analysis. No personally identifiable information (e.g., names, national identification numbers) was included in the dataset.

### Methodological limitations

As a hospital-based case series, this study has inherent limitations. The findings may not be generalizable to the wider population of Afghanistan, as they reflect the experience of a single tertiary referral center and may be subject to referral bias. Reliance on retrospectively collected secondary data may have resulted in incomplete documentation or misclassification for some variables, such as tobacco use or economic status. The absence of tumor staging data and long-term patient outcomes limits the clinical depth of the analysis. Furthermore, a substantial proportion of missing BMI data (38%) may affect the reliability of nutritional status assessment. These limitations are considered when interpreting the results.

## Results

During the study period, a total of 917 patients diagnosed with esophageal and gastric cancers were included in this hospital-based case series. Of these, 708 cases (77.2%) were diagnosed with esophageal cancer, while 209 cases (22.8%) had gastric cancer. The baseline demographic characteristics of the study population, including age, sex, marital status, ethnicity, and province of residence, are presented in Table 1.

**Table 1:** Distribution of demographic characteristics

Variables	Category	Esophageal cancer (*n*)	Esophageal cancer (%)	Gastric Cancer (*n*)	Gastric Cancer (%)
Age Group	15–24	5	0.7%	4	1.9%
	25–34	19	2.6%	5	2.3%
	35–44	81	11.4%	20	9.5%
	45–54	198	27.9%	48	22.9%
	55–64	200	28.2%	67	32%
	65–74	158	22.3%	53	25.3%
	> 75	47	6.6%	12	5.7%
	Total	708	100%	209	100%
Sex Distribution	Male	377	53.2%	149	71.2%
	Female	331	46.7%	60	28.8%
	Total	708	100%	209	100%
Marital Status	Married	595	84%	182	87%
	Single	5	0.7%	3	1.4%
	Widowed	108	15.2%	24	11.4%
	Total	708	100%	209	100%
Ethnicity	Pashtun	344	48.5%	91	43.5%
	Tajik	199	28.1%	74	35.4%
	Hazara	70	9.8%	28	13.3%
	Uzbek	60	8.4%	8	3.8%
	Turkmen	11	1.5%	2	0.9%
	Sadat	4	0.5%	3	1.4%
	Arab	5	0.7%	2	0.9%
	Pashai	4	0.5%	0	0%
	Baloch	3	0.4%	0	0%
	Nuristani	2	0.2%	0	0%
	Aimaq	1	0.1%	0	0%
	Bayat	0	0%	1	0.4%
	Qaderi	1	0.1%	0	0%
	Qizilbash	1	0.1%	0	0%
	Unknown	3	0.4%	0	0%
	Total	708	100%	209	100%
Provinces	Kabul	118	16.6%	63	30.1%
	Ghazni	64	9%	10	4.7%
	Helmand	43	6%	9	4.3%
	Takhar	40	5.6%	6	2.8%
	Kunduz	39	5.5%	2	0.9%
	Balkh	26	3.6%	11	5.2%
	Lugar	25	3.5%	9	4.3%
	Baghlan	26	3.6%	10	4.7%
	Wardak	25	3.5%	10	4.7%
	Badakhshan	22	3.1%	12	5.7%
	Faryab	28	3.9%	7	3.3%
	Zabul	29	4%	6	2.8%
	Parwan	13	1.8%	14	6.6%
	Laghman	17	2.4%	4	1.9%
	Nangarhar	23	3.2%	1	0.9%
	Bamian	10	1.4%	4	1.9%
	Daikundi	13	1.8%	3	1.4%
	Harat	13	1.8%	2	0.9%
	Uruzgan	16	2.2%	2	0.9%
	Kapisa	11	1.5%	3	1.4%
	Badghis	15	2.1%	2	0.9%
	Kandahar	15	2.1%	2	0.9%
	Paktia	6	0.8%	5	2.3%
	Farah	12	1.6%	2	0.9%
	Jawzjan	12	1.6%	1	0.4%
	Paktika	8	1.1%	3	1.4%
	Ghour	9	1.2%	0	0%
	Khost	9	1.2%	3	1.4%
	Samangan	8	1.1%	3	1.4%
	Sarepol	8	1.1%	0	0%
	Nimroz	2	0.2%	0	0%
	Kunar	1	0.1%	0	0%
	Noristan	2	0.2%	0	0%
	total	708	100%	209	100%

Regarding ethnic distribution, Pashtun and Tajik patients constituted the largest proportions in both esophageal and gastric cancers. This distribution reflects the overall ethnic composition of Afghan population, where Pashtuns and Tajiks form the majority, rather than indicating a higher susceptibility to these malignancies among these ethnic groups. Without population-based denominators, it is not possible to calculate ethnicity-specified incidence rates or draw conclusions about differential risk across ethnic groups.

Esophageal cancer had a relatively balanced distribution between males (53.2%) and females (46.8%), indicating that risk factors for this cancer in Afghanistan may affect both sexes equally. In contrast, gastric cancer was significantly more common in males (71.2%), which may reflect gander-based differences in exposure to risk factors such as smoking, occupational hazards, or dietary habits.

The age distribution indicated that both esophageal and gastric cancers predominantly affected middle-aged and older adults. The highest proportion of cases was observed among patients aged 55–64 years, followed by those aged 45–54 years and 65–74 years, while younger age groups constituted a relatively small proportion of the study population. A comparable age-related pattern was observed for both cancer types. Detailed age-specific distributions are shown in Table 2.

**Table 2:** Age-specific distribution of esophageal and gastric cancers

Age Group	Esophageal cancer *n* (%)	Gastric Cancer *n* (%)	Total Number of Cases (%)
15–24	5 (0.7%)	4 (1.9%)	9 (1%)
25–34	19 (2.6%)	5 (2.3%)	24 (2.6%)
35–44	81 (11.4%)	20 (9.5%)	101 (11%)
45–54	198 (27.9%)	48 (22.9%)	246 (26.8%)
55–64	200 (28.2%)	67 (32%)	267 (29.1%)
65–74	158 (22.3%)	53 (25.3%)	211 (23%)
> 75	47 (6.6%)	12 (5.7%)	59 (6.4%)
Total	708 (100%)	209 (100%)	917 (100%)

The socio-demographic and behavioral characteristics of the patients are summarized in Table 3.

**Table 3:** Socio-demographic and behavioral characteristics

Variables	Category	Esophageal cancer (*n*)	Esophageal cancer (%)	Gastric Cancer (*n*)	Gastric Cancer (%)
Occupation	Employed	31	4.3%	15	7.1%
	Self-employed	399	56.3%	84	40.1%
	Unemployed	274	38.7%	110	52.6%
	Unknown	4	0.56%	0	0%
	Total	708	100%	209	100%
Economic status^a^	Good	104	14.7%	54	34.8%
	Weak	604	85.3%	155	65.2%
	Total	708	100%	209	100%
Tobacco use^b^	Yes	377	53.2%	149	71.3%
	No	332	46.8%	60	28.7%
	Total	708	100%	209	100%
Chronic disease history^c^	Acute	0	0%	3	1.4%
	Chronic	708	100%	206	98.6%
	Total	708	100%	209	100%
Body Mass Index (BMI)^d^	Underweight	84	11.8%	31	14.8%
	Normal	268	37.8%	82	39.2%
	Overweight	70	9.8%	10	4.7%
	Obese	14	1.9%	10	4.7%
	Unknown	272	38.4%	76	36.3%
	Total	708	100%	209	100%

^a^Economic status: Physician’s assessment based on patient’s self-report

^b^Tobacco use: Defined as the use of tobacco products, including cigarettes, cigars, pipe tobacco, chewing tobacco, and naswar

^c^Chronic disease: Documented history of long-term conditions

^d^BMI: WHO categories; “Unknown” for missing data

Low economic status was documented in 85.3% of esophageal cancer patients and 65.2% of gastric cancer patients. Tobacco use was reported in 53.2% of esophageal and 71.3% of gastric cancer cases.

Regarding chronic disease history, all esophageal cancer patients (100%) and 98.6% of gastric cancer patients had documented chronic conditions. In the medical records, “chronic disease” includes a broad range of conditions such as diabetes mellitus (sugar), hypertension, ischemic heart disease, chronic respiratory diseases (asthma, COPD), and other non-communicable diseases. BMI data were available for 61.6% of esophageal and 63.7% of gastric cancer patients. Among those with available measurements, normal weight was the most frequent category.

All cases in this series were initially detected by ultrasound examination, which served as the primary diagnostic modality. Table 4 presents the initial diagnostic modalities, and Table 5 presents the final histopathological diagnosis.

**Table 4:** Initial diagnostic modalities

Cancer Type	Initial Diagnostic Finding	Number of Cases	Percentage (%)
Esophageal Cancer	Esophageal Mass	708	77.2%
Gastric Cancer	Stomach Mass	209	22.8%
Total		917	100%

**Table 5:** Final histopathological diagnosis

Cancer Type	Histological Subtype	Number of Cases	Percentage (%)
Esophageal Cancer	Squamous Cell Carcinoma (SCC)	496	70%
	Adenocarcinoma	125	17.6%
	Carcinoma	86	12.2%
	Lymphoma	1	0.1%
	Total	708	100%
Gastric Cancer	Adenocarcinoma	170	81.3%
	Carcinoid Tumor	36	17.2%
	Lymphoma	3	1.4%
	Total	209	100%

Histopathological examination showed that squamous cell carcinoma was the predominant subtype in esophageal cancer (70.0%), followed by adenocarcinoma (17.6%). In gastric cancer, adenocarcinoma was the most common subtype (81.3%), followed by carcinoma tumor (17.2%). Other types constituted smaller proportions.

The chi-square test demonstrated a statistically significant association between sex and cancer type (χ² = 20.75, df = 1, *p* < 0.001), with gastric cancer occurring more frequently among men. The detailed distribution is shown in Table 6.

**Table 6:** Association between sex and cancer type (Chi-square test)

Sex	Esophageal Cancer *n* (%)	Gastric Cancer *n* (%)	Total *n* (%)
Male	377 (53.2%)	149 (71.3%)	526 (57.4%)
Female	331 (46.8%)	60 (28.7%)	391 (42.6%)
Total	708 (100%)	209 (100%)	917 (100%)

Chi-square test: x^2^= 20.75, df = 1, *p* < 0.001

## Discussion

This hospital-based case series from Jumhoriat Hospital, Kabul, provides updated evidence on the pattern of esophageal and gastric cancers in Afghanistan. Among 917 patients, esophageal cancer (77.2%) was more common than gastric cancer (22.8%), with a ratio of approximately 3.4:1. This predominance of esophageal cancer aligns with previous hospital-based studies from Afghanistan [14, 15] and reflects the country’s location within the Central Asian Esophageal Cancer Belt. In contrast, population-based data fromm high-income countries often show a higher proportion of gastric cancer, highlighting regional heterogeneity in cancer epidemiology [16, 17].

The age distribution in our cohort showed a clear peak in the 55-64-year age group for both cancer types, with very few cases below 35 years. This pattern is consistent with international evidence indicating that upper gastrointestinal cancers typically develop after decades of exposure to risk factors [16, 18, 19] Comparable age trends have been reported in regional studies from Afghanistan [15, 20] and other low-resource setting in Africa and Asia [15, 19].

Sex distribution differed significantly between the two cancer types. Esophageal cancer showed a relatively balanced distribution between males (53.2%) and females (46.8%), whereas gastric cancer demonstrated a marked male predominance (71.2%) (x^2^ = 20.75, *p* < 0.001). This finding is consistent with global literature showing male predominance in gastric cancer [17, 20, 21]. The balanced sex distribution in esophageal cancer observed in our study has been reported in some high-risk regions, particularly where environmental and household exposures such as indoor air pollution, dietary habits, and food preparation practices affect both sexes equally [18, 19]. Studies from Afghanistan have similarly reported that risk factors for esophageal cancer, including hot tea consumption, oral snuff use, and low fruit intake, are prevalent in both men and women [15, 20].

Histopathological examination confirmed that squamous cell carcinoma (SCC) was the predominant subtype of esophageal cancer (70.0%), while adenocarcinoma was the dominant subtype of gastric cancer (81.3%). The predominance of esophageal SCC constitutes over 90% of cases. This pattern is consistent with studies from other high-incidence regions in Asia and Africa, where SCC remains the dominant histological type, in contrast to western countries where adenocarcinoma is increasingly common [16–18]. The predominance of gastric adenocarcinoma in our cohort is consistent with previous reports from Afghanistan [22] and other low-resource settings [20, 23].

Socioeconomic assessment showed that a substantial proportion of patients, particularly those with esophageal cancer (85.3%), belonged to low economic status. This finding is in agreement with studies linking upper gastrointestinal cancers to social deprivation, poor nutritional status, and limited access to preventive and diagnostic services [15, 19, 21]. The high prevalence of reported tobacco use, especially among gastric cancer patient (71.3%), warrants careful interpretation. In this study, the variable refers to documented tobacco use as recorded in the medical records. Previous case-control studies from Afghanistan have demonstrated significant associations between oral snuff use, hot tea drinking, and esophageal cancer [15, 20], supporting the biological plausibility of these findings.

Regarding chronic disease history, all esophageal cancer patient (100%) and 98.6% of gastric cancer patients had documented chronic conditions. This finding should be interpreted with caution, as it likely reflects routine documentation practices rather than true comorbidity burden. In the medical records, “chronic disease” encompasses a broad range of conditions including hypertension, diabetes mellitus, and chronic respiratory diseases, which are systematically recorded for nearly all admitted patients as part of standard history-taking. Additionally, the older age of the study population naturally increases the prevalence of such conditions.

### Health system implications

The finding of this study have several implications for cancer control in Afghanistan. First, the predominance of esophageal SCC highlights the need for primary prevention strategies targeting modifiable risk factors prevalent in the population, including tobacco use, hot tea consumption, and dietary practices. Public health education campaigns should focus on raising awareness of these risk factors, particularly in high-risk communities. Second, the advanced age at diagnosis (peak 55–64 years) and the high proportion of patients with low economic status suggest significant delays in diagnosis and barriers to healthcare access. Strengthening early detection capacity at primary and secondary care levels, particularly for symptomatic individuals, could improve outcomes.

 Third, the absence of population-based cancer registry data in Afghanistan limits the ability to monitor cancer trends and evaluate prevention efforts. Investment in cancer registry infrastructure is essential for evidence-based cancer control planning [24]. Finally, the high prevalence of tobacco use among cancer patients underscores the need for integrated tobacco prevention and cessation programs as part of comprehensive cancer control strategies.

## Limitations

This study has several limitations. First, as a hospital-based case series, the finding may not be generalizable to the wider Afghan population. Referral bias is likely, as Jumhoriat Hospital serves as a national referral center, and patients from provinces with better access to healthcare or higher socioeconomic status may be overrepresented. Second, the retrospective design and reliance on medical records may have resulted in incomplete documentation for some variables, particularly for behavioral risk factors such as tobacco use, which rely on patient self-report and may be subject to recall and social desirability biases. Third, data on tumor staging were not available, limiting the ability to assess disease severity at presentation and its implications for prognosis. Fourth, the high proportion of missing BMI data (approximately 38%) limits the assessment of nutritional status. Fifth, the study period (October 2023 to August 2024) is relatively short, and seasonal or annual variations in case presentation cannot be assessed. Sixth, the overrepresentation of Pashtun and Tajik patients reflects the general population composition of Afghanistan and should not be interpreted as evidence differential cancer risk; without population-based denominators, ethnicity-specific incidence rates cannot be calculated. Seventh, the high prevalence of documented chronic disease (100% in esophageal cancer patients) likely reflects documentation practices rather than true comorbidity burden, limiting the reliability of this variable. Finally, as a descriptive study, no causal inferences can be drawn regarding the associations observed.

## Conclusion

This hospital-based case series from Jumhoriat Hospital, Kabul, provides updated evidence on the demographic and histopathological patterns of esophageal and gastric cancers in Afghanistan. The predominance of esophageal squamous cell carcinoma and the high prevalence of modifiable risk factors-including tobacco use, hot tea consumption, and low fruit intake- highlight the need for targeted primary prevention strategies through public health education. Strengthening early detection capacity at primary care levels and establishing clear referral pathways could reduce diagnostic delays, particularly given the advanced age at diagnosis and high proportion of patients with low economic status. Investment in a population-based cancer registry is essential for effective cancer surveillance and evidence-based planning. Furthermore, integrated tobacco prevention and cessation programs should be incorporated into comprehensive cancer control strategies.

Further analytical studies, including case-control and cohort designs, are needed to better understand the etiological factors contributing to the high burden of these malignancies in Afghanistan and to inform evidence-based prevention and early detection efforts.

## Data Availability

The datasets used and/or analyzed during the current study are available from the corresponding author on reasonable request.
