# Correction: Hospital-based patterns of esophageal and gastric cancer cases at a major tertiary referral center in Kabul, Afghanistan: a retrospective case series

**DOI:** 10.1186/s12885-026-16269-8

**Published:** 2026-06-23

**Authors:** Mohammad Farid Tawakoli, Hamed Salihee, Abdulhafiz Rahmati, Mohammad Nasim Hatami

**Affiliations:** Public Health Faculty, Kabul University of Medical Sciences, Kabul, Afghanistan

**Correction: BMC Cancer 26**,** 591 (2026)**

**https://doi.org/10.1186/s12885-026-15945-z**

Following publication of the original article [1], the author requested to update the term "Drug use" to "Tobacco use". 

The original article has been updated.

## References

[1] Tawakoli MF, Salihee H, Rahmati A, et al. Hospital-based patterns of esophageal and gastric cancer cases at a major tertiary referral center in Kabul, Afghanistan: a retrospective case series. BMC Cancer. 2026;26:591. 10.1186/s12885-026-15945-z. 41906109 10.1186/s12885-026-15945-z PMC13159276

